# Paravascular spaces: entry to or exit from the brain?

**DOI:** 10.1113/EP087424

**Published:** 2019-01-09

**Authors:** Erik N. T. P. Bakker, Daphne M. P. Naessens, Ed VanBavel

**Affiliations:** ^1^ Amsterdam UMC University of Amsterdam Department of Biomedical Engineering and Physics Amsterdam Cardiovascular Sciences Amsterdam The Netherlands

**Keywords:** cerebral circulation, cerebrospinal fluid, glymphatic, interstitial fluid, pulsatility

## Abstract

**New Findings:**

- **What is the topic of this review?**
  In this symposium report, we review the glymphatic clearance from the brain.
- **What advances does it highlight?**
  Evaluation of the evidence indicates that cerebrospinal fluid flows along paravascular spaces at the surface of the brain. However, bulk flow along penetrating arteries into the brain, followed by exit along veins, requires further confirmation. Clearance from the brain, based on mixing, might provide an alternative explanation for experimental findings.

**Abstract:**

The interstitial fluid of the brain provides the environment for proper neuronal function. Maintenance of the volume and composition of interstitial fluid requires regulation of the influx and removal of water, ions, nutritive and waste products. The recently described glymphatic pathway might contribute to some of these functions. It is proposed that cerebrospinal fluid enters the brain via paravascular spaces along arteries, mixes with interstitial fluid, and leaves the brain via paravascular spaces along veins. In this symposium report, we review the glymphatic concept, its concerns, and alternative views on interstitial fluid–cerebrospinal fluid exchange.

## 1 INTRODUCTION

The brain interstitial fluid (ISF) needs to provide a healthy environment for neuronal function. It does so by the delivery of oxygen and nutrients from nearby capillaries and by the elimination of waste products in the opposite direction. Not all waste, however, can be eliminated via diffusion or specific transporters across the blood–brain barrier (BBB) into the bloodstream. Therefore, there is a need for additional mechanisms of ISF refreshment. In peripheral tissues, ISF is continuously renewed through transport of water and solutes from blood vessels. Most of the fluid is recycled back into the vessels at the venous side of the circulation, while the remainder is taken up by lymph vessels. The brain is different in this respect because far less fluid extravasates because of hydrostatic forces. This is a consequence of the very low permeability for ions such as sodium (Hladky & Barrand, 2016). Thus, osmotic pressure driving transvascular water transport involves not only the macromolecules, as in the classical Starling theory, but also the ions. Ionic osmotic pressure is orders of magnitude higher than colloid osmotic pressure, and any leakage of water owing to hydrostatic pressure would quickly be opposed. In addition, the brain is unique in the sense that it lacks a true lymphatic system. Thus, although it is of obvious importance that ISF homeostasis is maintained in terms of volume and composition, exactly how the brain accomplishes this is currently unclear and an area of ongoing research.

## 2 THE GLYMPHATIC THEORY

**Figure 1:**
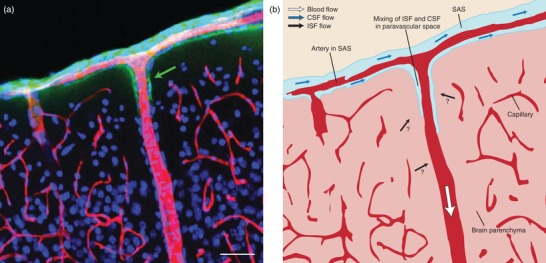
(a) Image of the paravascular space (green arrow) along a penetrating artery at the surface of a rat brain. Red, lectin staining to delineate the endothelium; green, staining of endogenous immunoglobulins present in the cerebrospinal fluid; and blue, DAPI nuclear stain. (b) Schematic diagram of the brain, with the flow patterns in the subarachnoid space, paravascular space and a penetrating artery. The amount of interstitial fluid that is formed and its contribution to local flow patterns are unknown. Abbreviations: CSF, cerebrospinal fluid; ISF, interstitial fluid; and SAS, subarachnoid space. Scale bar represents 50 μm

The work of Iliff et al. (2012) provided a conceptual framework for earlier observations by Cserr (Ichimura, Fraser, & Cserr, 1991) and others (Rennels, Gregory, Blaumanis, Fujimoto, & Grady, 1985) that could answer some of the unsolved questions regarding ISF homeostasis. Their glymphatic theory states that cerebrospinal fluid (CSF) enters the brain along paravascular spaces surrounding arteries (see Figure 1), passes through astrocyte endfeet in an aquaporin‐4 (AQ4)‐dependent manner, enters the parenchyma, where it mixes with ISF, and leaves the brain along venous paravascular spaces. The bulk flow through the parenchyma drags along waste products, including amyloid‐β, and impaired glymphatic function could therefore be important for neurodegenerative diseases, such as Alzheimer's disease. Subsequent studies from the same group complied with this view and suggested that the glymphatic pathway is impaired in a variety of conditions, including ageing (Kress et al., 2014), traumatic brain injury (Petraglia et al., 2014) and small infarcts (Wang et al., 2017), whereas it is more active during sleep and under general anaesthesia (Xie et al., 2013).

However, it is important to note that several aspects of the glymphatic theory have been challenged by theoretical considerations and experimental studies (for reviews, see Abbott, Pizzo, Preston, Janigro, & Thorne, 2018; Hladky & Barrand, 2014; Smith & Verkman, 2018). In our view, the most important unresolved issues are as follows: (i) the direction, if any, of flow in the paravascular space of penetrating vessels; (ii) the physical basis for the pressure gradients that drive paravascular and ISF flow; (iii) the role of pressure pulsations; and (iv) the contribution of the water‐selective channel, AQ4, to solute transport.

There are several potential caveats in the interpretation of data in this area of research. For instance, firstly, the infusion of tracers could easily overwhelm physiological mechanisms of solute dispersal, particularly when infused into the parenchyma. Secondly, rapid distribution of tracers does not prove net bulk flow, because mixing could achieve a similar pattern. Thirdly, modulation of paravascular flow by pressure pulsations does not necessarily mean that pulsations drive net flow. Lastly, CSF flow through paravascular channels on the surface of the brain does not provide evidence for flow around the penetrating vessels and into the parenchymal tissue.

## 3 THE PARAVASCULAR SPACE AND DIRECTION OF FLOW

The glymphatic theory proposes that inflow into the brain parenchyma occurs along arteries in the direction of blood flow. This was based on analysis of *in vivo* tracer spreading, using two‐photon imaging, and *ex vivo* fluorescence microscopy on brain slices. Using microsphere tracking, recent work from our group confirmed that at the brain surface, CSF flows along arteries in the direction of blood flow in a pulsatile manner (Bedussi, Almasian, de Vos, VanBavel, & Bakker, 2018). Further confirmation of this flow pattern in a non‐invasive manner was provided by the work of Harrison et al. (2018). Using novel magnetic resonance imaging sequences, these authors showed that CSF flows along the middle cerebral artery in rats. However, it is important to note that by no means does this imply that paravascular flow continues along penetrating arteries into the brain. As will be discussed later, alternative explanations exist for tracer spreading along arteries into the brain, such as mixing, rather than bulk flow. The influx of CSF along arteries, as proposed by Iliff et al. (2012), appears completely opposite to earlier findings from the group of Weller and Carare (Carare et al., 2008). In a series of papers, these authors reported efflux of tracers along arteries (for review, see Weller, Sharp, Christodoulides, Carare, & Mollgard, 2018), opposite to the direction of blood flow. This puzzling difference has not been resolved at present. However, we speculate that methodological differences could play an important role in reaching these opposing views. The group of Weller and Carare mostly relied on parenchymal injections of tracers and relatively early time points of post‐mortem analysis. In contrast, the group of Iliff and Nedergaard mostly injected tracers into the cisterna magna when visualizing specific distribution pathways. Possibly, the paravascular spaces around arteries represent low‐resistance pathways, in which the physiological direction of flow, or the absence of it, is easily overwhelmed by the pressure‐driven injection of a tracer. Data from our group substantiates this, because commonly used infusion rates of tracers clearly increase intracranial pressure (Bedussi, van derWel, et al., 2017). In addition, it should also be kept in mind that even apparently very small quantities of tracer solutions (≤1 μl) still represent a relatively large volume in the narrow extracellular space of a rodent brain. Thus, a volume of 1 μl equals a sphere with a diameter of 1.24 mm, which is certainly not small compared with the size of the rodent brain structures.

An alternative explanation for the discrepancy regarding the direction of paravascular flow around arteries is the possible existence of opposite flows, one in the paravascular space and one within the vessel wall, along the basement membranes of smooth muscle cells. Thus, there are different views on the exact anatomical structure through which tracers enter and leave the brain. Iliff et al. (2012) refer to ‘physically and anatomically’ separate channels for CSF flow when discussing *in vivo* observations obtained with two‐photon microscopy. Our data show that the paravascular spaces at the level of the meninges is continuous with the subarachnoid space (SAS) and appears as a widening created by meningeal blood vessels. This widening of the SAS is particularly large around arteries, probably because of the rounded shape in comparison to the more flattened veins, and can be observed in both humans and rodents (Bedussi et al., 2018). Weller and Carare refer to perivascular flow along basement membranes that starts at the level of the capillaries and travels upstream along arterial smooth muscle cells. In fact, in a recent study they propose that paravascular spaces might be a point of tracer entry, whereas perivascular outflow might occur along this anatomically separate compartment of basement membranes around the same artery (Albargothy et al., 2018). However, theoretical work suggests that the resistance in the narrow basement membrane compartment is far too high to allow bulk flow (Faghih & Sharp, 2018). In addition, this requires some anatomical basis for separate compartments. This could be represented by leptomeningeal sheets around arteries. However, recent electron microscopic images show that these sheets contain openings (stomata), which would effectively eliminate the separation into different compartments (Pizzo et al., 2018).

## 4 DRIVING FORCES FOR PARAVASCULAR FLOW

The glymphatic theory implies a pressure difference between the paravascular space around arteries and the paravascular space around veins in order to create bulk flow. However, it is unclear whether there is a physical separation of arterial and venous paravascular spaces at the level of the SAS, which is required to allow pressure differences to exist. In our hands, the paravascular spaces along arteries and veins appear continuous with the SAS (Bedussi, van derWel, et al., 2017). Such open communication would effectively eliminate potential pressure differences, which are necessary to drive glymphatic flow (Faghih & Sharp, 2018). In agreement with this view, recent work shows that even at very early time points, tracers are present in the paravascular spaces of both arteries and veins at the level of the SAS (Ma et al., 2018). Thus, it appears that these physical and anatomical features do not concur with the proposed bulk flow through the parenchyma according to the glymphatic theory.

## 5 PRESSURE PULSATIONS

Pressure pulsations have been repeatedly suggested to drive paravascular flow (Iliff et al., 2013; Schley, Carare‐Nnadi, Please, Perry, & Weller, 2006). We recently recorded relatively large pressure oscillations in the cisterna magna of rats induced by ventilation, and smaller oscillations induced by the heart beat (Naessens, de Vos, VanBavel, & Bakker, 2018). However, a fundamental problem with this hypothesis is that oscillations in vascular diameter that result from these pressure fluctuations require a valve in the paravascular space to generate bulk flow in this compartment. This is analogous to the generation of blood flow by the heart, which requires valves to operate effectively. At present, such valves have not been identified. In our opinion, the paravascular flow that is observed at the brain surface could be explained simply by the production of CSF, which leaves the ventricular system along the foramen of Magendie and flows in a rostral direction towards the cribriform plate along the brain surface. Thus, oscillations imposed by the ventilation and heart beat could modulate the flow pattern, but not necessarily drive net flow. Again, net flow at the brain surface does not provide evidence for inflow along paravascular spaces around arteries into the brain. In fact, recent work shows that tracers do not appreciably enter the brain parenchyma after injection into the cisterna magna and rapidly leave the brain via the cribriform plate and other perineural pathways (Ma et al., 2018). However, the oscillations in pressure in the CSF compartment could play a role in the transport of solutes along paravascular spaces of penetrating arteries. Thus, in the case of stagnant or very low net flow, pressure oscillations could induce mixing in these paravascular spaces. Theoretical work indicates that such mixing facilitates the efflux of waste from the parenchyma by accelerating ISF–CSF exchange (Asgari, de Zelicourt, & Kurtcuoglu, 2016).

## 6 AQUAPORIN‐4

The group of Verkman focused on the role of AQ4 and the presence of bulk flow in the parenchyma. Indeed, it is difficult to conceive how water‐selective AQ4 channels would facilitate solute and tracer influx and efflux, a key feature of the glymphatic concept. In an attempt to replicate the experiments with *Aqp4* knockout mice, these authors challenged the findings of Iliff and colleagues and concluded that tracer spreading in the parenchyma is determined by diffusion and is independent of AQ4 (Smith, Yao, Dix, Jin, & Verkman, 2017). Regarding AQ4, at present the data therefore remain inconclusive. Differences in experimental approaches, such as the type of anaesthesia and genetic background (CD1 *versus* C57Bl/6) of the animals, might interfere. In addition, the role of AQ4 is perhaps misinterpreted, because *Aqp4* knockout mice can develop astrocyte endfoot swelling (Zhou et al., 2008), which could reduce the space between astrocyte endfeet and, thereby, indirectly limit CSF–ISF interaction.

## 7 EFFECTS OF HYPERTENSION

Among other cardiovascular risk factors, hypertension has been described as an important contributor to cerebrovascular disease, leading to cognitive impairment and dementia (Iadecola et al., 2016). Studies are ongoing to unravel this complicated relationship, including some that address the question of whether antihypertensive treatment can prevent or delay dementia (Moll van Charante et al., 2016). Whether hypertension affects fluid flows in the brain is unclear. As described in the previous section, pressure in the CSF compartment oscillates, which could induce mixing in paravascular spaces along penetrating arteries (Bedussi, van derWel, et al., 2017). It is possible that elevated blood pressure and hypertension‐induced remodelling of small arteries and arterioles (Baumbach & Heistad, 1991) impair the oscillations in diameter that induce mixing. As a consequence, waste products, such as amyloid‐β, could remain in paravascular spaces and, with time, form aggregates around these vessels.

An alternative explanation for the impact of hypertension could be based on enhanced ISF formation and sieving of waste products at barriers between the ISF and CSF compartments. Several studies showed endothelial dysfunction in cerebral vessels of hypertensive animals (Baumbach & Heistad, 1988), which might affect the integrity of the BBB. We speculated that this could lead to increased ISF formation, a notion that was supported by increased spreading of tracers released into the hippocampus (Bedussi, Naessens, et al., 2017). However, more recent work from our group showed that the BBB and blood–CSF barrier function of spontaneously hypertensive rats is still intact with respect to the permeability to small solutes, such as fluorescein (Naessens et al., 2018). It is possible that the BBB dysfunction is very subtle in hypertensive rats, initially affecting ions and water only. Overt leakiness might be present only in specific areas, including the paraventricular nucleus and brainstem (Biancardi, Son, Ahmadi, Filosa, & Stern, 2014; Buttler et al., 2017; Setiadi, Korim, Elsaafien, & Yao, 2018). Nonetheless, there is a remarkable similarity between sites of tracer accumulation and sites of amyloid‐β deposition in rodents (Bedussi, Naessens, et al., 2017). This suggests that barriers formed by astrocytes, including those around paravascular spaces around arteries, can act as a sieve that allows water to pass from the ISF to the CSF, whereas larger solutes tend to accumulate.

## 8 CONCLUSIONS

There is strong evidence for the existence of paravascular spaces along arteries on the brain surface. Several studies have confirmed a pulsatile flow pattern in these channels, but further evidence is needed to prove whether or not pulsations drive bulk flow. Both experimental work and modelling studies are needed here. Paravascular spaces continue along penetrating arteries into the parenchyma, but the presence of (pulsatile) net flow herein is unclear. If net flow is absent or very low, mixing could facilitate CSF–ISF exchange in these extensions of the CSF compartment. Paravascular spaces around veins are present on the brain surface, but the evidence for paravenous efflux from the brain lacks confirmation. We speculate that leakage and/or secretion of water and solutes from capillaries contributes to the formation of ISF. The quantity of this fluid is likely to be relatively small in comparison to other tissues, owing to the restrictions provided by the BBB. Nonetheless, in the case of BBB dysfunction, this could provide a net outflow of ISF from the brain parenchyma to the CSF with sieving of larger solutes, such as amyloid‐β, at barriers formed by astrocytes, including those formed by astrocyte endfeet around arteries.

## COMPETING INTERESTS

None declared.
